# Identification of PAM4 (clivatuzumab)-reactive epitope on MUC5AC: A promising biomarker and therapeutic target for pancreatic cancer

**DOI:** 10.18632/oncotarget.2760

**Published:** 2015-01-19

**Authors:** Donglin Liu, Chien-Hsing Chang, David V. Gold, David M. Goldenberg

**Affiliations:** ^1^ IBC Pharmaceuticals, Inc., Morris Plains, New Jersey 07950, United States of America; ^2^ Immunomedics, Inc., Morris Plains, New Jersey 07950, United States of America; ^3^ Garden State Cancer Center, Center for Molecular Medicine and Immunology, Morris Plains, New Jersey 07950, United States of America

**Keywords:** MUC5AC, epitope mapping, PAM4, pancreatic cancer, cysteine-rich subdomain

## Abstract

PAM4 is a monoclonal antibody showing high specificity for pancreatic ductal adenocarcinoma (PDAC). Humanized PAM4 labeled with ^90^Y in combination with low-dose gemcitabine has shown promising therapeutic activity, and is being evaluated in a phase III clinical trial. Prior efforts have suggested that PAM4 potentially reacts with MUC5AC, a secretory mucin expressed *de novo* in early pancreatic neoplasia and retained throughout disease progression. In present study, we provide further evidence validating MUC5AC as the PAM4 antigen, and locate PAM4-reactive epitope within the N-terminal cysteine-rich subdomain 2 (Cys2), thus differentiating PAM4 from most anti-MUC5AC antibodies known to-date. Specifically, we show (i) PAM4-antigen and MUC5AC were co-localized in multiple human cancer cell lines, including Capan-1, BxPC-3, and CFPAC-1; (ii) MUC5AC-specific siRNA prominently reduced the expression of both MUC5AC and PAM4-antigen in CFPAC-1 cells; (iii) PAM4 preferentially binds to the void-volume fractions from Sepharose-CL2B chromatography of Capan-1 culture supernatants, which were revealed by Western blot to display the ladder pattern characteristic of oligomeric MUC5AC; and (iv) the N-terminal Cys2 within several recombinant MUC5AC fragments is essential for binding to PAM4. These findings shed light on the mechanism of PAM4-based diagnosis and treatment for pancreatic cancer, and guide further exploration of its clinical utility.

## INTRODUCTION

The number of patients who succumb to pancreatic ductal adenocarcinoma (PDAC) each year continues to rise, unlike other leading cancers where surveillance and/or screening technologies have led to a decrease in cancer-related mortality rates [1–3]. Unfortunately, the mortality rate for PDAC is nearly equal to the incidence. The overall survival rate for all stages of pancreatic cancer diagnosed between 2001 and 2007 is only 20% after one year, and about 6% after 5 years [3]. With the alarming increase in PDAC incidence, it is projected that by the year 2030, pancreatic cancer will become the second leading cause of cancer deaths in the United States [4]. The major reason for this poor prognosis is the inability to detect the disease at an early stage, when curative measures may have a greater opportunity to provide successful outcomes.

Biomarkers, whether they are biological, chemical, or physical in nature, have proven of significant value in providing information leading to the earlier detection and diagnosis of cancer, such as breast [5], colon [6], and prostate [7], resulting in improved patient outcomes. Unfortunately, this has not been the case for PDAC. Despite considerable attention directed towards discovery of biomarkers for PDAC [8], to date no FDA-approved means for early detection/diagnosis exists.

PAM4 is a murine monoclonal antibody (mAb) showing high specificity for PDAC compared with normal tissues and other cancers. At the tissue level, the reactivity of PAM4 is highly restricted to PDAC, with the biomarker expressed (or becomes accessible) at the earliest stages of neoplastic development [9–10], including pancreatic intraepithelial neoplasia (PanIN) and intraductal papillary mucinous neoplasm (IPMN). Notably, the PAM4-biomarker is absent from normal pancreas and benign, non-neoplastic lesions. In over 50 surgical specimens of chronic pancreatitis, the PAM4-biomarker was identified only within associated PanIN lesions and not by the inflamed parenchyma, including ducts, acinar cells, and acinar-ductal metaplasia [11].

Preclinical studies have demonstrated the potential applications of PAM4 for radioimmunoimaging and radioimmunotherapy of pancreatic carcinoma [12–13]. In patients, ^90^Y-labeled, humanized PAM4 (^90^Y-clivatuzumab tetraxetan, hereafter referred to as ^90^Y-hPAM4) was well tolerated with manageable hematologic toxicity under maximal tolerated ^90^Y dosing, and produced objective responses in both chemotherapy-naïve and -refractory patients with advanced PDAC [14]. Further, ^90^Y-hPAM4 in combination with low-dose gemcitabine showed enhanced therapeutic efficacy in patients with metastatic pancreatic cancer [15]. In a recently completed phase Ib study [16] involving 58 patients with metastatic PDAC who had at least 2 prior therapies, multiple cycles of fractionated ^90^Y-hPAM4 in combination with low radiosensitizing doses of gemcitabine significantly (*P* = 0.004) improved the Kaplan-Meier median overall survival of this difficult-to-treat (stage-4 disease) population to 7.9 months, compared to those receiving only ^90^Y-hPAM4 (3.4 months). These promising results led to the ongoing phase III registration trial of ^90^Y-hPAM4 in combination with gemcitabine (NCT01956812).

In addition, PAM4 or hPAM4-based ELISA has been devised and evaluated for detection of PDAC, showing that nearly two-thirds of patients having confirmed stage-1 disease had elevated PAM4 antigen in their serum [17–18]. However, the current assay, which employs hPAM4 as the capture antibody and a polyclonal rabbit anti-mucin antiserum (IgG fraction) as a probe, is not optimal, because the polyclonal probe is available in only limited quantities and, more importantly, is not itself specific for the PAM4 antigen. Another concern for further development of the assay has been the unknown nature of the antigen marker to which PAM4 is reactive. Given the clinical merit and ongoing evaluation of hPAM4 as a potential diagnostic and therapeutic agent for PDAC, there is an urgent need to identify the PAM4 epitope. Towards this end, we recently proposed [19] that PAM4 was reactive with the human MUC5AC, a polymeric gel-forming mucin with the monomeric form consisting of more than 5,000 amino acid residues organized into three major regions [20]: a signal peptide and four von Willebrand factor (vWF)-like cysteine-rich domains (D1, ≈D2, D' and D3) in the N-terminal region, a MUC11p15-type domain preceding the heavily O-glycosylated mucin domain in the central region, and a cluster of vWF-like cysteine-rich domains (D4, B, C, and CK) in the C-terminal region. In addition, 9 cysteine-rich subdomains (designated Cys1, Cys2, Cys3, Cys4, Cys5, Cys6, Cys7, Cys8, and Cys9) are interspersed within the mucin domain. Herein we present further evidence to support MUC5AC as the PAM4-reactive mucin and, importantly, have mapped the PAM4 epitope to Cys2.

## RESULTS

### Co-localization of the hPAM4 antigen and MUC5AC in different cell lines

**Figure 1:**
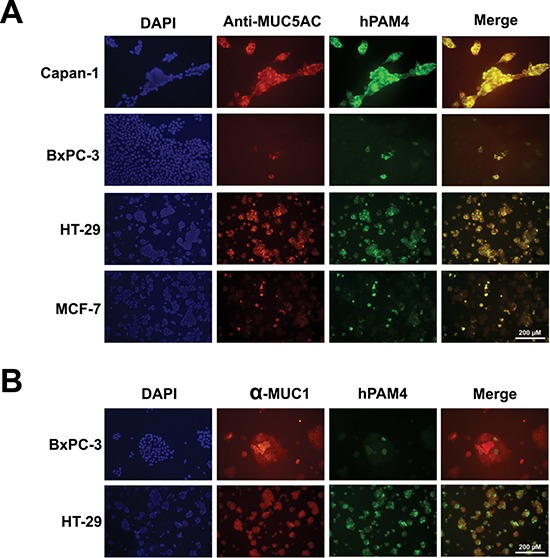
Co-localization of PAM4 antigen with MUC5AC by immunofluorescence staining **(A)** Mucin-expressing cell lines were stained with DAPI, hPAM4, and anti-MUC5AC (2-12M1 for Capan-1 and BxPC-3; 2-11M1 for HT-29 and MCF-7), then examined by immunofluorescence microcopy. **(B)** BxPC-3 and HT-29 cells were stained with DAPI, hPAM4, and α-MUC1. PAM4 antigen was shown to co-localize with MUC5AC, not MUC1.

Several cell lines were subjected to immunofluorescence microscopy in order to evaluate localization patterns (heterogeneous and/or homogenous) of MUC1, MUC5AC, and/or MUC17, as detected by hPAM4 and other mucin-specific mAbs. The cell lines examined included those derived from human pancreatic (Capan-1, BxPC3, CFPAC-1, and AsPC-1), colorectal (HT-29 and LS174 T), breast (MCF-7), and lung (A549) carcinomas. As shown in Figure 1 and Supplementary Table S1, in each of the cell lines examined, hPAM4 exclusively co-localized with MUC5AC (as identified by two anti-MUC5AC mAbs, 2-11M1 and 2-12M1, Figure 1A), but not with MUC1 (Figure 1B) or MUC17 (data not shown), suggesting that MUC5AC is the hPAM4-reactive antigen.

### Co-knockdown of the hPAM4 antigen and MUC5AC by MUC5AC-specific SiRNA

**Figure 2:**
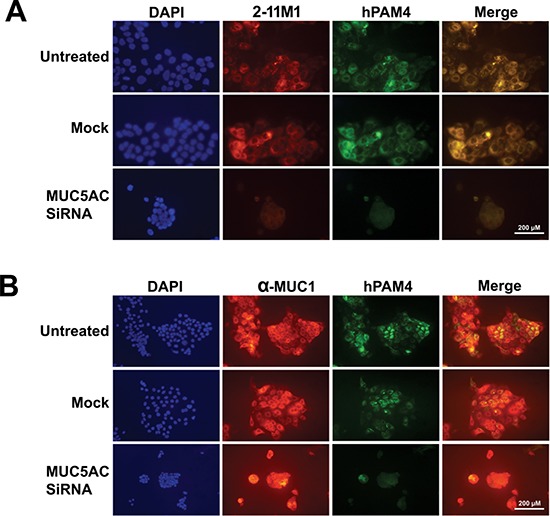
Co-knockdown of PAM4 antigen and MUC5AC by MUC5AC-specific siRNA **(A)** CFPAC-1 cells were treated with a MUC5AC-specific siRNA, followed by staining with DAPI, hPAM4, and 2-11M1. **(B)** CFPAC-1 cells were treated with a MUC5AC-specific siRNA, followed by staining with DAPI, hPAM4, α-MUC1. Untreated Cells or cells treated with only the transfection agent (mock) served as controls. Cells treated with MUC5AC-specific siRNA lost the binding to anti-MUC5AC and hPAM4 concurrently, with little effect on the binding to anti-MUC1.

The disparate localization between PAM4 and anti-MUC1 or anti-MUC17 indicates that PAM4 reacts with neither MUC1 nor MUC17. On the other hand, the co-localization of PAM4 and the two anti-MUC5AC mAbs (2-11M1 and 2-12M1) is consistent with PAM4 being specific for MUC5AC [19]. To investigate if hPAM4 associates with MUC5AC, we employed the RNAi method to specifically knockdown MUC5AC. As shown in Figure 2A, hPAM4 and 2-11M1 are co-localized in untreated CFPAC-1 cells, as well as the mock-treated (transfection agent alone) cells. In contrast, treatment with MUC5AC-specific siRNA resulted in substantially reduced immunostaining for both 2-11M1 and hPAM4. Moreover, as shown in Figure 2B, siRNA knockdown of MUC5AC did not alter the anti-MUC1 immunostaining, providing further evidence that hPAM4 is not reactive with MUC1.

### Presence of the hPAM4 antigen in the culture supernatant of mucin-producing carcinoma cell lines

**Figure 3:**
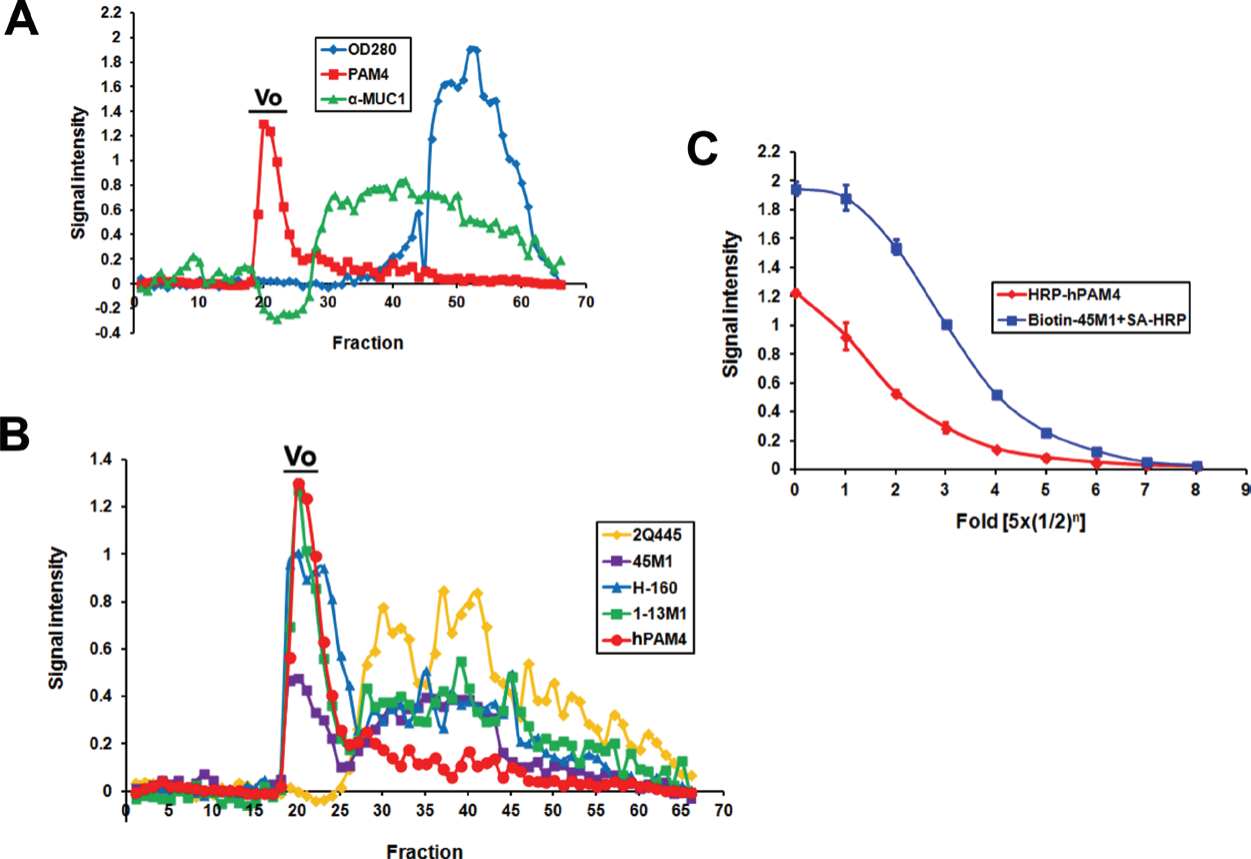
Immunoreactivity of fractions eluted from Sepharose CL-2B **(A)** Capan-1 cell culture supernatant was separated on a Sepharose CL2B column with the eluted fractions analyzed by hPAM4 and α-MUC1. **(B)** The void-volume (Vo) fractions of Capan-1 reacted positively with three anti-MUC5AC antibodies (45M1, 1-13M1 and H-160), but not with 2Q445, which recognizes the unglycosylated tandem repeat region of MUC5AC. **(C)** The Capan-1 void-volume peak, following capture by 2-11M1, could be detected directly by HRP-hPAM4, or indirectly by biotin-45M1 plus SA-HRP.

MUC5AC is a highly oligomeric secretory mucin that has been isolated from cell culture and *in vivo* mucous secretions [21–22]. Our early studies showed that hPAM4 reacts with mucin derived from the Capan-1 xenografted human PDAC [9]. In the current study, we used Sepharose® CL-2B molecular sieve chromatography to separate the mucin species secreted into the supernatant of Capan-1. The eluted fractions were then examined for immunoreactivity with hPAM4 and α-MUC1. As shown in Figure 3A, PAM4-reactive substance was present predominantly in the void-volume peak, whereas only subsequently eluted fractions were found reactive with α-MUC1. When the Capan-1 void-volume peak was probed with anti-MUC5AC antibodies, we found a positive response with 45M1, 1-13M1, and H-160, but not 2Q445, as shown in Figure 3B. It is noted that the void-volume peaks obtained from other cancer cell lines known to secret MUC5AC, such as HT-29 [21], LS 174T [23], SW1990 [24], CFPAC-1 [25], and Calu-3 [26], were all tested positive for reactivity with hPAM4 (Supplementary Figure S1).

Direct evidence that correlates the hPAM4-reactive substance in the Capan-1 void-volume peak with MUC5AC is provided by a sandwich ELISA formatted to quantify the MUC5AC captured by 2-11M1, which reacts with the N-terminal domains of MUC5AC [27]. As shown in Figure 3C, 2-11M1-captured MUC5AC could be detected by hPAM4 in a dose-dependent manner, demonstrating that hPAM4 binds to a different region of MUC5AC from 2-11M1. The additional results obtained with 45M1, which serves as a positive control, also support the previous conclusions that the epitopes of 45M1 [28] and 2-11M1 on MUC5AC are non-overlapping.

### Electrophoretic resolution of the hPAM4-reactive void-volume fractions on agarose gel

**Figure 4:**
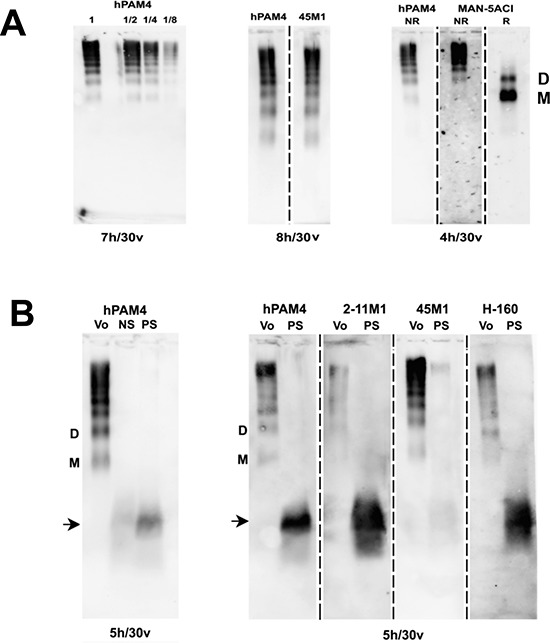
Agarose gel electrophoresis **(A)** The Capan-1 void-volume peak displayed the characteristic banding pattern of MUC5AC as revealed by Western blot analysis with hPAM4, 45M1, and MAN-5ACI. In the left panel, samples in the lanes marked as 1, 1/2, 1/4, and 1/8 were tested undiluted, 2-, 4- and 8-fold diluted, respectively. In the far right panel, the monomeric and dimeric MUC5AC were indicated as M and D, respectively. **(B)** The serum from a pancreatic cancer patient (PS) tested positive for hPAM4-reactive substance was differentially detected by hPAM4 and three anti-MUC5AC antibodies (2-11M1, 45M1, and H-160). The Capan-1 void-volume peak (Vo) and normal serum sample (NS) were included as controls.

To further verify the hPAM4-reactive substance to be MUC5AC, the Capan-1 void-volume peak was separated by electrophoresis on 0.7% agarose gel, and subsequently probed with hPAM4 (Figure 4A, left panel), 45M1 (Figure 4A, middle panel), or MAN-5ACI (Figure 4A, right panel) by Western blotting. Under non-reducing conditions, a group of bands resembling the ladder-like pattern reported for MUC5AC [21, 29] was clearly discerned by all three antibodies. In contrast, under reducing conditions, two bands were revealed by MAN-5ACI, but undetectable by either hPAM4 or 45M1, which corroborate the previous findings that the predominant fast-migrating band and the minor band trailing behind represent the MUC5AC monomer and a reduction-resistant dimer, respectively [21], and that neither 45M1 [28] nor hPAM4 [9] should react with a reduced mucin.

### Detection of hPAM4-reactive substance in serum samples of pancreatic cancer patients

The visualization of MUC5AC by hPAM4 as a characteristic ladder in Western blot following agarose gel electrophoresis prompted us to examine whether such a pattern could be demonstrated for patient serum found positive with the presently formulated PAM4-based assay [16, 17]. As shown in Figure 4B, a broad band migrating faster than MUC5AC monomer was detected by hPAM4 and several MUC5AC-specific antibodies, such as 2-11M and H-160, but not by 45M1, suggesting the PAM4-reactive antigen in the patient serum could be derived from an immature MUC5AC variant, or a breakdown product of mature MUC5AC.

### Mapping of the hPAM4 epitope on MUC5AC

**Figure 5:**
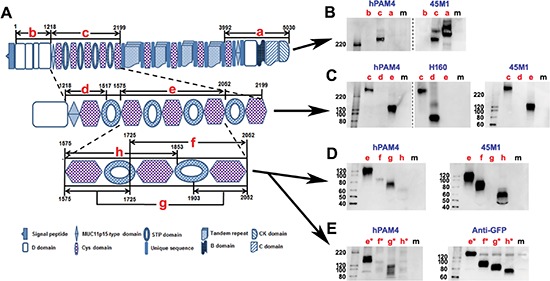
Mapping the PAM4-reactive epitope on human MUC5AC **(A)** Schematic diagram of different MUC5AC recombinant fragments (a–h) generated in PANC-1 cells for mapping PAM4 epitope; Numbers are AA positions in the MUC5AC protein sequence (UniProtKB/Swiss-Prot: P98088). **(B to E)** Western blot of various MUC5AC recombinant fragments by hPAM4, anti-MUC5AC, or anti-GFP antibodies, as indicated. See text for details. Lane **m** indicates samples from untransfected cells.

The disparity in the reactivity of hPAM4 and 2Q445 with the Capan-1 void-volume peak, as noted in Figure 3B, suggests that the hPAM4 epitope is not in the tandem repeat region of MUC5AC recognized by 2Q445 [30]. Therefore, we excluded the tandem repeat region (AA2199-3992) and decided to express in PANC-1 cells three large recombinant fragments (designated as a, b, and c) that comprise the remainder of MUC5AC (Figure 5A). We found that hPAM4 did not react with the C-terminal a-fragment (AA3992-5030) or the N-terminal b-fragment (AA1-1217), suggesting its epitope was located outside the N-terminal D1-D2-D'-D3 domains and the C-terminal region encompassing Cys9-D4-B-C-CK domains. In contrast, the c-fragment (AA1218-2199), which spans the five N-terminal cysteine-rich subdomains (Cys1-2-3-4-5), reacted with hPAM4 as shown by Western blot (Figure 5B, left panel). Expectedly, the c-fragment was found to react also with 1-13M1 (data not shown) and 45M1 (Figure 5B, right panel), which recognize cysteine-rich subdomains of class-2 (Cys2 and Cys4) and class-3 (Cys3, 5, 6, 7, 8 and 9), respectively. We next expressed two sub-fragments (d and e) within the c-fragment and showed (Figure 5C, left panel) that hPAM4 failed to react with the d-fragment (AA1218-1517) comprising 11P15-Cys1, but strongly stained the e-fragment (AA1575-2052) comprising Cys2-3-4. We then expressed three overlapping sub-fragments (f, g, and h) of the e-fragment and showed (Figure 5D, left panel) that hPAM4 stained the g-fragment (AA1575-1725 joined to AA1903-2052, comprising Cys2 and Cys4 with Cys3 deleted), but barely the f-fragment (AA1725-2052, comprising Cys3-4) or the h-fragment (AA1575-1853, comprising Cys2-3). The differential reactivity of hPAM4 observed for the e-, f-, g-, and h-fragments was confirmed (Figure 5E, left panel) with the respective GFP-fused counterparts (the e*-, f*-, g*- and h*-fragments); the expression of each was clearly shown by Western blot with anti-GFP (Figure 5E, right panel). Together, these results indicate that (i) the hPAM4 epitope resides within the e-fragment, which contains the Cys2-3-4 region; (ii) the presence of Cys2 or Cys4, or both, is needed for recognition by hPAM4; (iii) Cys3 is essential for the binding of 45M1, since it stained each of the c- (Figure 5B, right panel; Figure 5C, rightmost panel), e-, f-, and h-fragments (Figure 5C, rightmost panel; Figure 5D, right panel), all of which contains Cys3; but not the g-fragment (Figures 5D, right panel), which lacks Cys3; and (iv) the validity of the d-fragment was supported by its positive staining with H-160 (Figure 5C, middle panel), whose epitope was reported to reside in AA1214-1373 [31] contained in the d-fragment.

**Figure 6:**
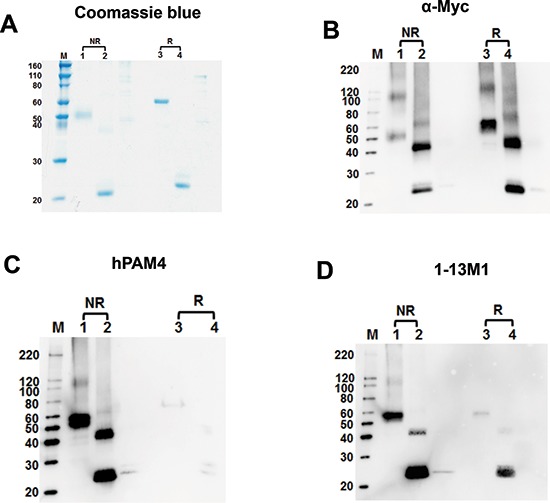
SDS-PAGE and Western blot analyses of recombinant MUC5AC fragments expressed in *E. coli* Four gels were run under similar conditions of SDS-PAGE. **(A)** One gel was stained with coomassie blue. The other three gels were transferred onto nitrocellulose membrane and stained with anti-Myc, hPAM4, and 1-13M1, in **(B**, **C**, and **D)** respectively. Samples, either reduced (R) or non-reduced (NR), were loaded at 500 ng/well; lane M, markers; lanes 1 & 3, Cys2-3-4 (AA1575-2052); lanes 2 &4, Cys2+ (AA1575-1725).

The successful expression of Cys2-3-4 (AA1575-2052) and Cys2+ (AA1575-1725) in *E. coli*, as evidenced by the coomassie blue staining (Figure 6A) and Western blot using anti-Myc (Figure 6B), was instrumental in further defining the location of the hPAM4 epitope to the Cys2 domain. The unglycosylated Cys2-3-4 and Cys2+ were isolated predominantly as monomeric species of 55.4 and 20.5 kDa, respectively. As shown in Figure 6C, hPAM4 reacts with non-reduced, but not the reduced, Cys2-3-4 and Cys2+. Although 1-13M1 also targets Cys2 or Cys4, its binding to both non-reduced and reduced Cys2+ (Figure 6D) differentiates it from hPAM4. Thus, we further establish that the hPAM4 epitope, being reduction-sensitive, is conformational, located within the Cys2 domain, and unlikely involving carbohydrates. We speculate that the weakly positive bands observed for hPAM4 in lanes 3 and 4 of Figure 6C could result from reformation of the disulfide bond to a varying degree in the process of blotting, which would restore the hPAM4 epitope.

## DISCUSSION

In the past decade, concerted efforts in the search of biomarkers for PDAC have produced compelling evidence that mucins are aberrantly expressed in this devastating malignancy, and have diverse biological functions in tumor development, progression, metastasis, and drug resistance [32]. Moreover, a number of studies [32–34] have shown that both cell-tethered and secreted mucins display different expression profiles in pancreatic cancer when compared to normal pancreas. As a *de novo* mucin in pancreatic cancer, MUC5AC could be detected as early as the pre-malignant/dysplastic stages [35], and was identified in a high percentage of PDAC [34, 36–37]. Our own endeavors [9–19] for over 20 years have focused on the exploration of mucin-reactive PAM4 as a potential diagnostic and therapeutic agent for PDAC. Although we have recently proposed MUC5AC to be the PAM4 antigen [19], the identification of the PAM4 epitope has lagged behind its clinical development, mainly due to the challenges encountered in characterizing MUC5AC, which is polymeric, heavily O-glycosylated, and present in several variant forms [38–40]. In the current study, we provide additional evidence from immunocytochemistry, RNA interference, and biochemical studies that authenticates MUC5AC as the hPAM4 antigen; and more importantly, have located the PAM4 epitope to the N-terminal region comprising Cys2 through the recombinant expression of MUC5AC domains [41]. We should note that DEGYTFCESPR, one of the 6 MUC5AC peptides most frequently detected in the pancreatic cystic lesions with malignant potential, and not in the benign lesions, is located in the Cys2 and Cys4 subdomains, as reported in a very recent study of mucin proteomics [42].

Based on their sequence similarity [43], the 9 Cys subdomains of MUC5AC have been characterized [40] as Class I (Cys1), Class II (Cys 2, Cys4; 98% identical), and Class III (Cys3, Cys5-9; 96% identical). Whereas each subdomain contains about 110 amino acid residues, including 10 remarkably conserved cysteine residues involved in intramolecular disulfide bonds, there is only one potential O-glycosylation site and no potential N-glycosylation site. These structural features appear to match the characteristics of the hPAM4 epitope. Earlier work [9] showed that the reactivity between PAM4 and its mucin antigen was negatively affected by heating, reduction of disulfide bonds, or certain protease digestion, suggesting that the PAM4 epitope is a conformational glycopeptide. While we have confirmed that reduced MUC5AC no longer reacts with hPAM4, the results obtained from the unglycosylated Cys2-3-4 and Cys2+ of this study also indicate that the hPAM epitope is retained under denaturing conditions (or can be readily restored following blotting or immobilization and washing), and unlikely to involve carbohydrates. Further experiments are in progress to determine whether hPAM4 recognizes a continuous or discontinuous epitope in the Cys2 subdomain of MUC5AC, as secreted by PDAC and its precursor lesions. Because Cys2 and Cys4 are 98% identical in amino acid sequence, including all of the 10 conserved cysteine residues, we expect the hPAM4 epitope is present on Cys4 also.

It is worthy of note that among the various anti-MUC5AC antibodies with mapped epitopes, which include the mouse mAbs of the M1 series: 1-13M1 [27], 2-11M1 [27], 9-13M1 [27], 19M1 [44], 21M1 [44], 62M1 [44], 45M1 [28], and 2-12M1 [28]; other murine mAbs such as CLH2 [45], SOMU1[27], 2Q445 [29], and NPC-1C [46]; and two rabbit polyclonal antibodies, H-160 [31] and MAN-5ACI [39], 1-13M1 is the only mAb reported to react with Cys2/4 domains of MUC5AC. Our data, however, indicate that 1-13M1 binds to a reduction-insensitive epitope, thus being different from that of hPAM4.

Because the Cys2, Cys3 and Cys4 subdomains are flanked by threonine/serine/proline (TSP)-rich sequences, which contain numerous O-glycosylation sites, we further note that the accessibility of hPAM4 to its epitope on Cys2 (or Cys4) could be masked by the surrounding oligosaccharides either structurally or in a conformation-dependent manner, or both. As such, hPAM4 would prevail for MUC5AC with specific decoration, which is mostly produced in PDAC, including the early-stage pancreatic cancer precursors, and occasionally occurs in other epithelial cancers [9].

In conclusion, we have located the hPAM4 epitope to the N-terminal Cys2 of MUC5AC and characterized it as a reduction-sensitive, carbohydrate-free epitope, whose access may be restricted by the surrounding oligosaccharides in the flanking TSP-domains. We believe the ultimate delineation of the hPAM4 epitope may lead to its exploration as a candidate for vaccine development, while providing valuable insight for diagnosis and treatment of MUC5AC-expressing cancers, such as biliary tract cancer [47], colorectal cancer [48], and gastric cancer [49], in addition to PDAC.

## MATERIAL AND METHODS

### Antibodies and reagents

Humanized PAM4 (hPAM4) was provided by Immunomedics, Inc. Horseradish peroxidase (HRP)-hPAM4 conjugate was generated using the SureLINK HRP Conjugation Kit (Kirkegaard & Perry Laboratories). MAN-5ACI, a rabbit antiserum against MUC5AC [39], was a generous gift from Dr. David J. Thornton (University of Manchester). Commercially available antibodies acquired include the following: four mouse mAbs against MUC5AC (45M1, 2-11M1, 2-12M1, and 1-13M1) from Thermo Fisher Scientific, one mouse monoclonal (2Q445) and one rabbit polyclonal (H-160) antibodies against MUC5AC from Santa Cruz Biotechnology, one mouse mAb against human MUC1 (MAB6298, α-MUC1) from R&D Systems, one rabbit polyclonal antibody against MUC17 (HPA031634) from Sigma-Aldrich, one rabbit polyclonal antibody against full-length GFP (α-GFP) from Clontech Laboratories, one rabbit polyclonal Myc-tag antibody (α-Myc) from Cell Signaling Technology, one FITC-labeled goat anti-human IgG (FITC-GAH) from Jackson ImmunoResearch Laboratories, and one Cy3-labeled goat anti-mouse IgG (Cy3-GAM) from EMD Millipore. The MUC5AC double-strand siRNA targeting sequence 5′-GGAGCCTGATCATCCAGCA-3′ was synthesized by GenScript. Sepharose^®^ CL-2B was purchased from Sigma-Aldrich.

### Cell culture

All cell lines were obtained from the American Type Culture Collection (ATCC) and have been authenticated by Promega using Short Tandem Repeat (STR) analysis. BxPC-3, HT-29, LS174T, MCF-7, and Calu-3 were grown in RPMI 1640 medium (Life Technologies) with 10% fetal bovine serum (FBS, Thermo Scientific HyClone); Capan-1 was grown in RPMI 1640 medium with 20% FBS; CFPAC-1 was grown in ATCC-formulated Iscove's Modified Dulbecco's Medium (IMDM) with 10% FBS; SW1990 was grown in ATCC-formulated Leibovitz's L-15 Medium with 10% FBS; and PANC-1 was grown in Dulbecco's Modified Eagle Medium (Life technologies) plus 10% FBS. All cell lines were incubated at 37 °C in 5% CO_2_ except SW1990, which was cultured in 100% air.

### Immunocytochemistry

Cells were plated on 8-chamber slides (Thermo Fisher Scientific) at approximately 2 × 10^4^ cells/chamber and incubated overnight at 37°C. Following removal of the medium, cells were fixed in 4% formalin (Sigma-Aldrich) for 15 min at RT, and then treated with 0.1% Triton X-100 in PBS for another 15 min. After washed twice with PBS, cells were incubated with 10 μg/ml of hPAM4 and a murine mAb against MUC5AC, α-MUC1, or a rabbit polyclonal antibody against MUC17 in PBS plus 1% BSA for 45 min at RT. Afterwards, cells were washed twice and incubated with a mixture of FITC-GAH and Cy3-GAM or Cy3-GAR in PBS plus 1% BSA for 30 min at RT. After three washes, chambers were dissembled. Slides were mounted with an antifade solution (VectaShield, Vector Laboratories) containing the nuclear counterstain, 4, 6-diamidino-2-phenylindole (DAPI). Image acquisition and analyses were performed using an Olympus fluorescence microscope with a Kodak camera system.

### RNA interference

CFPAC-1 cells grown to 90% confluence were used for transfection. MUC5AC siRNA or PBS alone (Mock) was 1:100 diluted into Opti-MEM I Medium (Life Technologies) prior to the addition of 1/100 volume of Lipofectamine® RNAiMAX Reagent (Life Technologies). After 20 min incubation at RT, the siRNA or Mock mixture was dispersed onto 8-chamber slides (80 μl/chamber). Meanwhile, cells were trypsinized, washed, diluted in complete growth medium, and then added at 8 × 10^3^ cells/400 μl/chamber. The final RNA concentration is 15.6 nM in a total volume of 480 μl. After 48 h incubation, cells were stained with hPAM4 and 2-11M1 (anti-MUC5AC) or α-MUC1 and examined by fluorescence microscopy as described above.

### Gel chromatography of cell culture supernatant

Capan-1 cells were cultured for 3-4 days to reach over 90% confluence. The spent media were collected, mixed with an equal volume of 8 M guanidine hydrochloride (GdmCl) in 20 mM sodium phosphate buffer (pH 7), and 10-fold concentrated using the Amicon ultrafiltration membrane with 30 kDa normal molecular weight limit (EMD Millipore). Gel chromatography was performed on a Sepharose CL-2B column (78 cm×2.6 cm) using 4 M GdmCl as the eluent and a flow rate of 40 ml/h. Fractions of 8 mL were collected and each analyzed for reactivity with hPAM4, 45M1, and α-MUC-1 by ELISA as follows. Briefly, MaxiSorp 96-well plates (Nunc, Roskilde, Denmark) were coated with CL-2B-eluted fractions (100 μl/well) at 37°C overnight, washed twice with PBS, and blocked with Casein Blocking Buffers (Thermo Fisher Scientific) for 1 h. HRP-hPAM4, 45M1, or α-MUC1 was diluted in PBS and added at 100 μl/well. After 1 h incubation at RT, plates with α-MUC1 were washed and incubated further with HRP-GAM for 1 h. Plates were washed and bound HRP-hPAM4 or HRP-GAM was detected with o-phenylenediamine dihydrochloride (0.4 mg/ml) in PBS plus 0.03% hydrogen peroxide as a substrate. The optical density was read at 490 nm using the EnVision 2100 Multilabel Reader (PerkinElmer). The fractions eluted in the void-volume peak were also pooled, dialyzed against the PBS-AG buffer (35.2 mM Na_2_H PO_4_.7H_2_O; 0.4 M NaCl; 6.5 mM NaH_2_PO4.H_2_O; 150 mM arginine; 150 mM monosodium glutamate, pH 8.0), and concentrated with 30 kDa Amicon Ultra centrifugal filters (EMD Millipore) for further analysis.

### MUC5AC sandwich ELISA

MaxiSorp 96-well plates were coated with 100 μl of 2-11M1 (20 μg/ml) in PBS and incubated at 4°C overnight. After blocking with casein buffer, a 5-fold concentrated void-volume peak pooled from the CL-2B fractionation of Capan-1 supernatant (hereafter referred to as the Capan-1 void-volume peak) was 2-fold serially diluted and added to the plate at 100 μl/well. After overnight incubation at RT, plates were washed and detected by HRP-PAM4, or by Biotin-45M1 plus HRP-streptavidin as a positive control.

### Agarose gel electrophoresis

Agarose gel electrophoresis was performed as described [21], with modifications. Briefly, the Capan-1 void-volume peak was concentrated in PBS-AG buffer and diluted with gel running buffer (40 mM Tris-acetate/1mM EDTA, 0.1% SDS, pH 8.0). In selective experiments, serum samples from normal subjects or pancreatic cancer patients enrolled in institutional review board–approved clinical trials were mixed with an equal volume of 8 M guanidine hydrochloride (GdmCl) and dialyzed into gel running buffer. All samples were supplemented with 1 M urea, 3% glycerol and 0.02% bromophenol blue before loading into thin wells shaped with a 0.8 mm-thick comb in 0.7% agarose gel (5.7 cm x 8.3 cm). Electrophoresis was performed at 30 V for 4 to 8 h in the Horizon 58 Electrophoresis Apparatus (LABRepCo).

### Construction of expression vectors for MUC5AC recombinant fragments

The *pSM-MUC5AC-CH-long* expression vector [28, 50], which encodes a signal sequence, a Myc tag (EQKLISEEDL), the human MUC5AC (Swiss-Prot accession no. P98088) C-terminal cysteine-rich part (AA3993-5030, a-fragment), and a histidine tag, was kindly provided by Dr. Gunner Hansson of Gothenburg University (Gothenburg, Sweden). Additional vectors were constructed from *pSM-MUC5AC-CH-long* by replacing the DNA sequence of AA3993-5030 with that of AA1-1217, AA1218-2199, AA1218-1517, AA1575-2052, AA1725-2052, AA1575-1723/1903-2052, AA1575-1853, and AA1575-1725, to express D1-D2-D'-D3 (b-fragment), 11P15-Cys1-2-3-4-5 (c-fragment), 11P15-Cys1 (d-fragment), Cys2-3-4 (e-fragment), Cys3-4 (f-fragment), Cys2/4 (g-fragment), Cys2-3 (h-fragment), respectively, as listed in Table 1. In addition, four GFP-fused fragments were produced by replacing the Myc tag with a full GFP sequence in the vectors encoding Cys2-3-4, Cys3-4, Cys2/4, and Cys2-3, resulting in the e*-, f*-, g*- and h*-fragment, respectively. Myc-tagged Cys2-3-4 and Cys2+ (i-fragment) DNA sequences were inserted into the pET26b vector (EMD Millipore) for expression in *E. coli* cells (Table 1).

**Table 1:** Recombinant MUC5AC fragments

Fragment	Tag	MUC5AC domains	AA # (P98088^a^)	MW^b^ (Da)	Expression	
					PANC-1	*E. coli*
**a**	Myc	Cys9-D4-B-C-CK	3992–5030	116,140	**+**	
**b**	Myc	D1-D2-D'-D3	1–1217	136,727	**+**	
**c**	Myc	11P15-Cys1-2-3-4-5	1218-2199	109,380	**+**	
**d**	Myc	11P15-Cys1	1218–1517	35,704	**+**	
**e**	Myc	Cys2-3-4	1575–2052	56,444	**+**	**+**
**f**	Myc	Cys3-4	1725–2052	39,686	**+**	
**g**	Myc	Cys2/4	1575–1725/1903–2052	37,171	**+**	
**h**	Myc	Cys2-3	1575–1853	35,452	**+**	
**I**	Myc	Cys2+	1575–1725	20,504		**+**
e*	GFP	Cys2-3-4	1575–2052	81,742	**+**	
f*	GFP	Cys3-4	1725–2052	64,983	**+**	
g*	GFP	Cys2/4	1575–1725/1903–2052	62,468	**+**	
h*	GFP	Cys2-3	1575–1853	60,749	**+**	

a UniProt;

b based on the unglycosylated protein.

### Expression of recombinant MUC5AC fragments

One day prior to transfection, PANC-1 cells were seeded in a 24-well plate at 2×10^5^/well and held at 37°C overnight. Transfection was performed using Lipofectamine 2000 (Life Technologies) with and without the recombinant plasmid DNA of interest. After 72 h, the spent media were collected and analyzed by Western blot following gel electrophoresis. To produce unglycosylated proteins, Myc-tagged Cys2-3-4 and Cys2+ fragments were also expressed in *E. coli* and purified from the inclusion body using HIS-Select Nickel Affinity Gel (Sigma-Aldrich), and refolded.

### Western blot

Samples were electrophoresed in the same gel or different gels under the same conditions. After electrophoresis, samples were transferred (100V, 1 h) onto a nitrocellulose membrane using the Mini Trans-Blot® cell system (Bio-Rad Laboratories) and probed with hPAM4, an anti-MUC5AC antibody, α-GFP, or α-Myc, as indicated. The signals were developed with SuperSignal™ West Dura Chemiluminescent Substrate (Thermo Fisher Scientific).

## SUPPLEMENTARY FIGURE AND TABLE


