# Supplementary material for: Identification of PAM4 (clivatuzumab)-reactive epitope on MUC5AC: A promising biomarker and therapeutic target for pancreatic cancer
Source: Oncotarget. 2015 Jan 19;6(6):4274–85. doi: 10.18632/oncotarget.2760 (PMC4414189; doi:10.18632/oncotarget.2760)
Supplement: Supplementary file 1 [file oncotarget-06-4274-s001.pdf]

SUPPLEMENTARY FIGURE AND TABLE

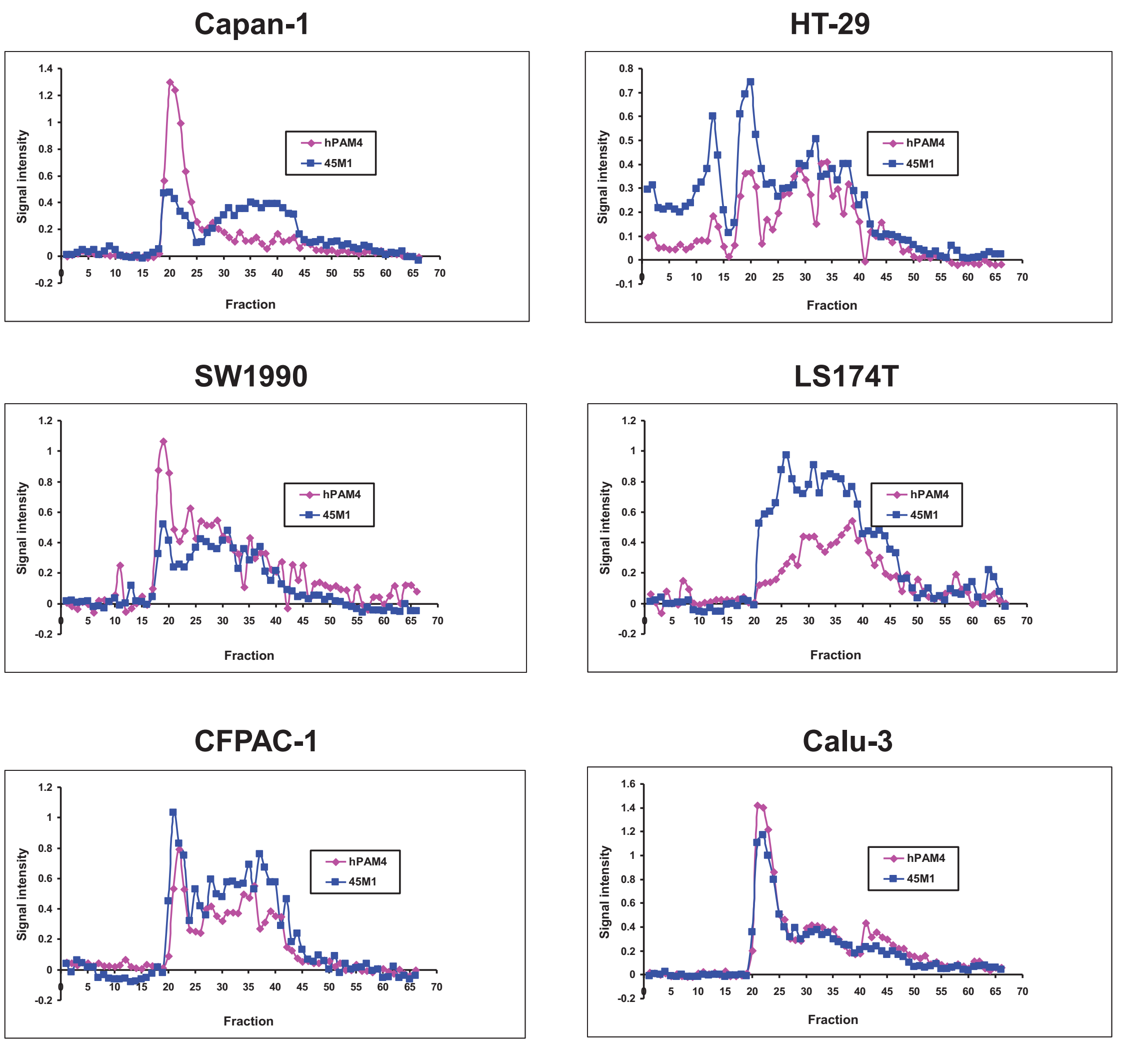

Supplementary Figure S1: Chromatography profiles of PAM4 antigen in different cell-culture media. The spent medium from each cell line was mixed with an equal volume of 8M GdmCl and 10-fold concentrated prior to gel chromatography on Sepharose CL-2B. Fractions were analyzed by ELISA for reactivity with hPAM4 and 45M1 respectively.

Supplementary Table S1: Immunostaining of various human cancer cell lines by hPAM4, anti-MUC5AC, anti-MUC1, and anti-MUC17

Cell line	Intensity[a]			
	hPAM4[b]	Anti-MUC5AC[b]	Anti-MUC1[c]	Anti-MUC17[c]
Capan-1	++++	++++	++++	+
AsPC-1	++	++	ND	+++
BxPC-3	+	+	++	ND
PANC-1	–	–	–	–
CFPAC-1	+++	+++	++++	ND
HT-29	+++	+++	++	ND
LS174T	+++	+++	++	ND
A549	++	+	–	ND
SK-MES-1	++	ND	ND	ND
MCF-7	++	++	+++	ND
HCT116	–	–	–	ND

[a]Very high, ++++; high, +++; medium, ++; low, +; negative, –.
[b]PAM4 antigen and MUC5AC were completely co-localized in Capan-1, AsPC-1, BxPC-3, CFPAC-1, HT-29, and MCF-7, and mostly co-localized in A549 and LS174T cell lines.
[c]PAM4 antigen was not co-localized with either MUC1 or MUC17 in all cell lines examined.
